# Relationship between very low low-density lipoprotein cholesterol concentrations not due to statin therapy and risk of type 2 diabetes: A US-based cross-sectional observational study using electronic health records

**DOI:** 10.1371/journal.pmed.1002642

**Published:** 2018-08-28

**Authors:** QiPing Feng, Wei-Qi Wei, Cecilia P. Chung, Rebecca T. Levinson, Alexandra C. Sundermann, Jonathan D. Mosley, Lisa Bastarache, Jane F. Ferguson, Nancy J. Cox, Dan M. Roden, Joshua C. Denny, MacRae F. Linton, Digna R. Velez Edwards, C. Michael Stein

**Affiliations:** 1 Division of Clinical Pharmacology, Department of Medicine, Vanderbilt University Medical Center, Nashville, Tennessee, United States of America; 2 Department of Biomedical Informatics, Vanderbilt University Medical Center, Nashville, Tennessee, United States of America; 3 Division of Rheumatology, Vanderbilt University Medical Center, Nashville, Tennessee, United States of America; 4 Vanderbilt Genetics Institute, Vanderbilt University Medical Center, Nashville, Tennessee, United States of America; 5 Vanderbilt Epidemiology Center, Institute for Medicine and Public Health, Vanderbilt University, Nashville, Tennessee, United States of America; 6 Division of Cardiovascular Medicine, Department of Medicine, Vanderbilt University Medical Center, Nashville, Tennessee, United States of America; 7 Department of Pharmacology, Vanderbilt University, Nashville, Tennessee, United States of America; Stanford University, UNITED STATES

## Abstract

**Background:**

Observations from statin clinical trials and from Mendelian randomization studies suggest that low low-density lipoprotein cholesterol (LDL-C) concentrations may be associated with increased risk of type 2 diabetes mellitus (T2DM). Despite the findings from statin clinical trials and genetic studies, there is little direct evidence implicating low LDL-C concentrations in increased risk of T2DM.

**Methods and findings:**

We used de-identified electronic health records (EHRs) at Vanderbilt University Medical Center to compare the risk of T2DM in a cross-sectional study among individuals with very low (≤60 mg/dl, *N =* 8,943) and normal (90–130 mg/dl, *N =* 71,343) LDL-C levels calculated using the Friedewald formula. LDL-C levels associated with statin use, hospitalization, or a serum albumin level < 3 g/dl were excluded. We used a 2-phase approach: in 1/3 of the sample (discovery) we used T2DM phenome-wide association study codes (phecodes) to identify cases and controls, and in the remaining 2/3 (validation) we identified T2DM cases and controls using a validated algorithm. The analysis plan for the validation phase was constructed at the time of the design of that component of the study. The prevalence of T2DM in the very low and normal LDL-C groups was compared using logistic regression with adjustment for age, race, sex, body mass index (BMI), high-density lipoprotein cholesterol, triglycerides, and duration of care. Secondary analyses included prespecified stratification by sex, race, BMI, and LDL-C level. In the discovery cohort, phecodes related to T2DM were significantly more frequent in the very low LDL-C group. In the validation cohort (*N =* 33,039 after applying the T2DM algorithm to identify cases and controls), the risk of T2DM was increased in the very low compared to normal LDL-C group (odds ratio [OR] 2.06, 95% CI 1.80–2.37; *P <* 2 × 10^−16^). The findings remained significant in sensitivity analyses. The association between low LDL-C levels and T2DM was significant in males (OR 2.43, 95% CI 2.00–2.95; *P <* 2 × 10^−16^) and females (OR 1.74, 95% CI 1.42–2.12; *P =* 6.88 × 10^−8^); in normal weight (OR 2.18, 95% CI 1.59–2.98; *P =* 1.1× 10^−6^), overweight (OR 2.17, 95% CI 1.65–2.83; *P =* 1.73× 10^−8^), and obese (OR 2.00, 95% CI 1.65–2.41; *P =* 8 × 10^−13^) categories; and in individuals with LDL-C < 40 mg/dl (OR 2.31, 95% CI 1.71–3.10; *P =* 3.01× 10^−8^) and LDL-C 40–60 mg/dl (OR 1.99, 95% CI 1.71–2.32; *P <* 2.0× 10^−16^). The association was significant in individuals of European ancestry (OR 2.67, 95% CI 2.25–3.17; *P <* 2 × 10^−16^) but not in those of African ancestry (OR 1.09, 95% CI 0.81–1.46; *P =* 0.56). A limitation was that we only compared groups with very low and normal LDL-C levels; also, since this was not an inception cohort, we cannot exclude the possibility of reverse causation.

**Conclusions:**

Very low LDL-C concentrations occurring in the absence of statin treatment were significantly associated with T2DM risk in a large EHR population; this increased risk was present in both sexes and all BMI categories, and in individuals of European ancestry but not of African ancestry. Longitudinal cohort studies to assess the relationship between very low LDL-C levels not associated with lipid-lowering therapy and risk of developing T2DM will be important.

## Abstract 2

Qiping Feng and colleagues highlight the association between low LDL-C concentrations and the risk of developing type 2 diabetes for European, but not African ancestry, patients not taking statins.

## Author summary

**Why was this study done?:**

- Lipid-lowering drugs (statins) are effective for prevention of coronary artery disease.
- Statin therapy is associated with increased risk of new-onset type 2 diabetes mellitus (T2DM).
- The risk of diabetes could be increased by statins directly or, alternatively, by low low-density lipoprotein cholesterol (LDL-C) concentrations.
- Available genetic studies suggest that low LDL-C concentrations are a risk factor for T2DM.
- This hypothesis has not been tested in a clinical setting without statin treatment.

**What did the researchers do and find?:**

- We used the de-identified electronic health records at Vanderbilt University Medical Center.
- We identified individuals with low LDL-C levels (less than 60 mg/dl) and with normal LDL-C levels (between 90 and 130 mg/dl). All LDL-C levels were in absence of statin treatment.
- We compared the risk of T2DM between these 2 groups.
- We found that individuals with low LDL-C levels had higher T2DM risk compared to those with normal LDL-C levels.
- This increased risk was present in both sexes and all body mass index categories, and in individuals of European ancestry but not in individuals of African ancestry.

**What do these findings mean?:**

- The findings suggest that special attention should be paid to risk of T2DM with therapies that lower LDL-C markedly.

## Introduction

Drugs such as HMG-CoA reductase inhibitors (statins) lower low-density lipoprotein cholesterol (LDL-C) concentrations and are effective for primary and secondary prevention of coronary artery disease [1–3]. However, statin therapy is associated with an approximately 9%–12% increase in the risk of new-onset type 2 diabetes mellitus (T2DM) [4,5]. The risk of diabetes could be increased by statins directly, and, in keeping with this possibility, the risk could be increased with higher doses [6]; however, genetic approaches have also implicated low LDL-C concentrations as a risk factor for T2DM.

Mendelian randomization studies using functional *HMGCR* variants as genetic instruments found a higher risk of T2DM in individuals with variants associated with lower LDL-C concentrations [5]. Also, variants in other genes associated with lower LDL-C concentrations, such as variants in *proprotein convertase subtilisin/kexin type 9 (PCSK9)*, were also associated with increased risk of diabetes [7,8]. Since PCSK9 and HMGCR are involved in lipid metabolism through distinct molecular pathways, the altered glycemic effect associated with variants in both genes is likely to be the result of their common effect on LDL-C concentrations.

Despite the findings from statin clinical trials and genetic studies, there is little direct evidence implicating low LDL-C concentrations in increased risk of T2DM. The available data from clinical trials and epidemiologic studies suffer from either small sample size or short follow-up. The relationship between low LDL-C concentrations and T2DM is important because new lipid-lowering medications can reduce LDL-C to extremely low levels [9]; however, little is known about the potential adverse effects of such long-term very low LDL-C levels.

Individuals who have very low LDL-C concentrations not due to lipid-lowering therapy can provide insights into the relationship between low LDL-C concentrations and T2DM. Here, we used de-identified electronic health records (EHRs) to test the hypothesis that very low LDL-C concentrations are associated with T2DM.

## Methods

### Data source

Data for this cross-sectional observational study were obtained from the Synthetic Derivative, which contains a de-identified copy of the EHR for every patient in the Vanderbilt University Medical Center system. This de-identified EHR is scrubbed of all Health Insurance Portability and Accountability Act (HIPAA) identifiers. New clinical data are added as they are generated. The Synthetic Derivative currently contains the de-identified records of >2.5 million unique individuals. It incorporates diagnostic and procedure codes, demographics, clinical care notes, patient history, problem lists, laboratory values, and medications, from which researchers can extract phenotypes such as disease diagnoses and treatment outcomes. [10–12]. Therefore, the Synthetic Derivative represents the population from a large teaching hospital and its ancillary services.

The study was approved by the Vanderbilt University Medical Center Institutional Review Board (IRB# 150137).

### Study population

**Fig 1:**
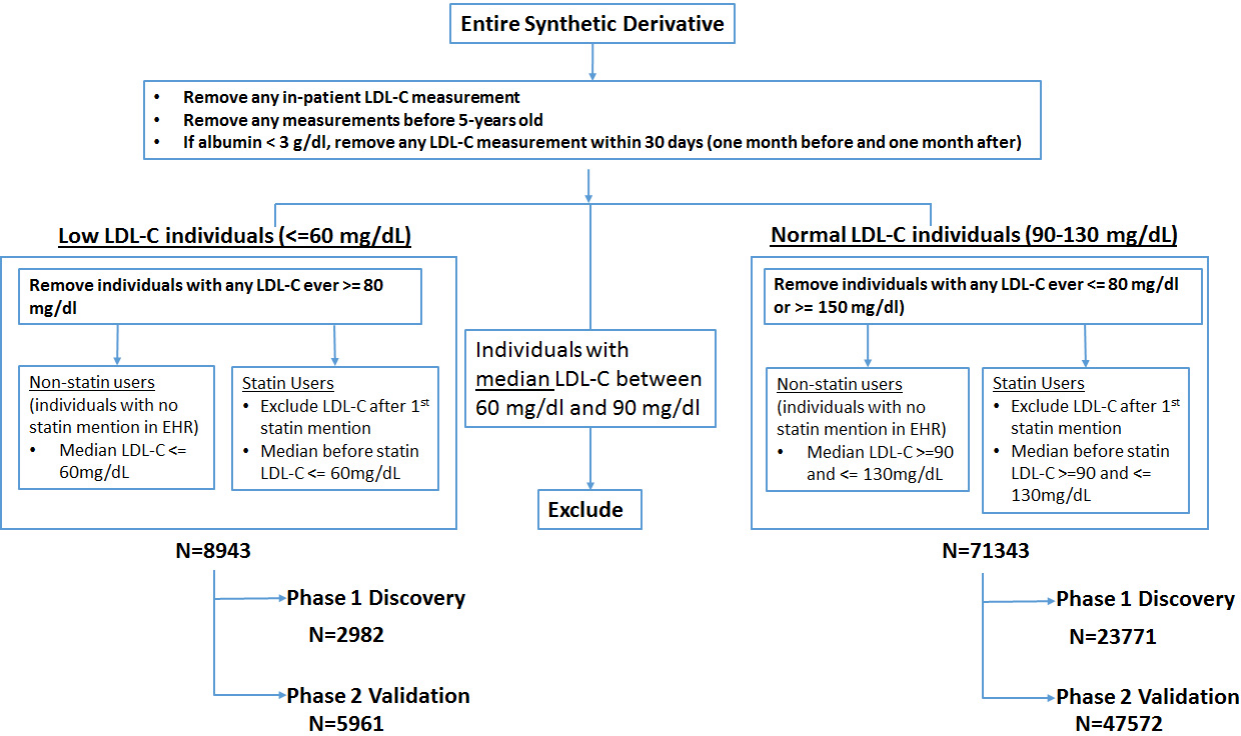
Algorithm to identify individuals with low or normal LDL-C. EHR, electronic health record; LDL-C, low-density lipoprotein cholesterol.

Patients in the Synthetic Derivative who had 1 or more outpatient LDL-C measurements were eligible to enter the cohort. LDL-C measurements were excluded if they were performed (1) in hospital, (2) before 5 years of age, (3) within 30 days of a serum albumin level < 3 g/dl (30 g/l), or (4) after the first mention of a statin in the EHR (Fig 1). The very low LDL-C cohort was defined as individuals with median LDL-C level ≤ 60 mg/dl (1 mg/dl = 0.02586 mmol/l) and no LDL-C measurement ever ≥80 mg/dl; the normal LDL-C cohort was defined as those with median LDL-C level between 90 mg/dl and 130 mg/dl and no LDL-C measurement ever ≥150 mg/dl or ≤80 mg/dl. We excluded individuals with median LDL-C concentrations between 60 and 90 mg/dl to reduce misclassification between the very low (hereafter “low”) and normal LDL-C cohorts. We manually reviewed the charts of 97 individuals with LDL-C concentrations less than 20 mg/dl to ensure that they were not receiving statin therapy. We removed individuals who had a median LDL-C < 0 mg/dl (*N =* 8) (on review these were found to be an artifact of the Friedewald formula for calculating LDL-C).

### Two-phase study design

We randomly divided the group into 1/3 for phase 1 (discovery) and 2/3 for phase 2 (validation). From previous experience, we knew that the EHR-based algorithm for T2DM would exclude some of the cohort who could not reliably be determined to be either a case or a control (e.g., individuals without a blood glucose measurement); thus, we assigned 2/3 of the cohort to the validation study to maintain statistical power.

#### Phase 1: Discovery

We used an efficient but not highly specific screening strategy previously developed by Denny et al. to facilitate rapid genotype–phenotype association studies using EHRs [12,13]. Specifically, International Classification of Disease–9th revision (ICD-9) codes were grouped into phecodes, which aggregate similar ICD-9 codes into a hierarchical system of more than 1,800 diseases, signs, and symptoms. Each custom phecode also has an associated control group that excludes other related conditions [12–14]. We tested all 6 T2DM-related phecodes (each of them representing an aspect of T2DM that is encountered in clinical practice), including 250.2, 250.21, 250.22, 250.23, 250.24, and 250.25 (S1 Table). Individuals with phenotype data were assigned status as a case, a control, or an exclusion for each of the phecodes. To be a case an individual had to have 2 or more ICD-9 codes for that phecode on different days. Individuals who had a single ICD-9 code for a phecode were excluded for the test on that code. All remaining individuals who were neither cases nor excluded were defined as controls. We used version 1.2 of the phecode definitions (available from http://phewascatalog.org).

#### Phase 2: Validation and fine phenotyping

To validate phase 1 observations, we applied a highly specific EHR-based algorithm to identify cases of T2DM and extracted covariates from the EHR. The algorithm to define T2DM was previously developed, validated, and implemented within the eMERGE network and had a positive predictive value > 98% in 3 EHR-based datasets [15,16] (https://www.phekb.org/phenotype/type-2-diabetes-mellitus).

Covariates including age, sex, race, body mass index (BMI), EHR length, and high-density lipoprotein cholesterol (HDL-C) and triglyceride concentrations were extracted from the EHR. Specifically, we calculated median values for BMI and HDL-C and triglyceride concentrations for each individual. Age was defined as the age at the most current ICD-9 code assignment, and EHR length was calculated from the date of the first available record to the date of the most current record. In the Synthetic Derivative, race is observer-reported; this approach correlates well with approaches using self-reported race or genetic information [17,18].

### Statistical analysis

We planned not to proceed beyond the discovery phase unless at least 1 of the phecodes for diabetes was significantly different in the 2 groups. The analysis plan for the validation phase was constructed at the time of the design of that component of the study and included adjustment for age, race, sex, BMI, HDL-C, triglycerides, and length of EHR. Secondary analyses with stratification by race, sex, BMI, and LDL-C were preplanned. Sensitivity analyses were planned after the results of the main analysis were known in order to test the validity of those findings.

#### Phase 1: Discovery

We tested the association between low LDL-C and 6 diabetes phenotypes (code 250.2 group) using logistic regression with adjustment for age, race, and sex using the PheWAS R package [19]. Results were adjusted for multiple testing, and *P*-values less than 0.0083 were considered significant.

#### Phase 2: Validation

For the primary analysis, we estimated odds ratios (ORs) and 95% confidence intervals (CIs) for the risk of having T2DM with adjustment for age, race, sex, BMI, length of EHR, and median HDL-C and triglyceride concentrations. We performed several secondary analyses including prespecified stratification by sex, race, BMI, and LDL-C level (S1 Fig) and post hoc sensitivity analyses restricting the analysis to those individuals with (1) at least 2 LDL-C measurements; (2a) no evidence of a previous medical condition that could be an indication for statin therapy (diabetes or myocardial infarction) and additionally (2b) no evidence of previous advanced renal disease or organ transplantation and additionally (2c) no evidence of previous stroke, transient cerebral ischemia, or peripheral vascular disease; (3) no evidence of receiving ezetimibe; and (4) excluding individuals who were <18 years old at the time of their median LDL-C value. Previous medical conditions were defined as conditions present up to 30 days after the first qualified LDL-C measurement. The presence of a potential indication for statin use was defined by the presence of any code in phecode groups 250.* (type 1 or 2 diabetes) or 411.* (ischemic heart disease). Advanced renal disease was defined as the presence of phecode 585.31, 585.32, or 585.34 or an estimated glomerular filtration rate of 29 ml/min or less. Organ transplantation was defined as the presence of ICD-9 code V42.0, V42.1, V42.2, V42.5, V42.6, V42.7, V42.81, or V42.82. Stroke, transient cerebral ischemia, and peripheral vascular disease were defined as the presence of ICD-9 code 434.91, 334.9, 435, 435.8, or 435.9. The use of ezetimibe was identified by MedEx, which processes clinical records to recognize medications [11]. Logistic regression analyses were performed using R. Interaction analyses were performed using R and STATA 14.2, and results are expressed as ORs with 95% CIs.

## Results

We identified 8,943 individuals with low (≤60 mg/dl) and 71,343 individuals with normal (90–130 mg/dl) LDL-C concentrations. Compared to the normal LDL-C group, individuals with low LDL-C were younger, had lower BMI and triglycerides, and were more likely to be female and of African ancestry (Tables 1 and S2). The normal and low LDL-C groups had similar sex distribution, HDL-C levels, and length of EHR. The discovery group was composed of 2,982 individuals with low and 23,771 with normal LDL-C concentrations; the validation group had 5,961 individuals with low and 47,572 with normal LDL-C (Fig 1).

**Table 1:** Demographic characteristics.

Characteristic	Category	Low LDL-C cohort	Normal LDL-C cohort
***Discovery (phase 1)***			
***N***		2,982	23,771
**Sex**	**Female**	1,613 (54.1%)	13,739 (57.8%)
	**Male**	1,367 (45.8%)	10,007 (42.1%)
**Race**	**European ancestry**	1,830 (61.4%)	16,628 (70.0%)
	**African ancestry**	623 (20.9%)	3,089 (13.0%)
	Other*	556 (18.6%)	4,054 (17.1%)
**Age (years)**		33.2 (19.5–51.8) (*N =* 2,940)	42.8 (30.1–55.4) (*N =* 23,377)
**BMI (kg/m**^**2**^**)**		24.5 (21.0–30.3) (*N =* 2,508)	27.1 (23.4–32.0) (*N =* 19,871)
**Lipid panel**	**LDL-C (mg/dl)**	52.0 (44.0–57.0)	109.0 (100.0–119.0)
	**Triglycerides (mg/dl)**	88.0 (58.0–151.0) (*N =* 2,949)	101.0 (72.0–148.0) (*N =* 23,575)
	**HDL-C (mg/dl)**	51.0 (38.5–66.0) (*N =* 2,942)	51.0 (41.0–63.0) (*N =* 23,597)
**Length of EHR (years)**		6.5 (2.3–11.6) (*N =* 2,940)	6.9 (2.5–11.9) (*N =* 23,376)
***Validation (phase 2)***			
***N***		5,961	47,572
**Sex**	**Female**	3,253 (54.6%)	27,392 (57.6%)
	**Male**	2,698 (45.3%)	20,140 (42.3%)
**Race**	**European ancestry**	3,744 (62.8%)	33,333 (70.1%)
	**African ancestry**	1,208 (20.3%)	6,226 (13.1%)
	Other*	1,009 (16.9%)	8,013 (16.8%)
**Age (years)**		33.2 (19.0–51.3) (*N =* 5,864)	42.6 (30.0–55.4) (*N =* 46,726)
**BMI (kg/m**^**2**^**)**		24.5 (20.9–30.0) (*N =* 5,032)	27.1 (23.4–32.1) (*N =* 20,128)
**Lipid panel**	**LDL-C (mg/dl)**	52.0 (44.0–57.0)	109.0 (100.0–119.0)
	**Triglycerides (mg/dl)**	88.0 (57.0–148.0) (*N =* 5,868)	102.0 (72.0–149.0) (*N =* 23,828)
	**HDL-C (mg/dl)**	51.0 (39.0–66.0) (*N =* 5,857)	51.0 (41.0–63.0) (*N =* 23,845)
**Length of EHR (years)**		6.6 (2.3–12.0) (*N =* 5,863)	6.9 (2.6–11.9) (*N =* 46,726)

Data are *N* (percent) or median (interquartile range). LDL-C and HDL-C: 1 mg/dl = 0.0256 mmol/l; triglycerides: 1 mg/dl = 0.0113 mmol/l.

*The category “Other” includes Asians, Pacific Islanders, and Native Americans as well as individuals whose race was not known.

HDL-C, high-density lipoprotein cholesterol; LDL-C, low-density lipoprotein cholesterol.

### Discovery cohort

There was a significant association between low LDL-C and 5 of the 6 T2DM phecodes in the phase 1 discovery dataset, and after adjusting for age, sex, and race, 4 of the 6 remained significantly associated (S3 Table). Compared to the normal LDL-C group, individuals with low LDL-C (≤60 mg/dl) were more likely to have codes for 250.2 (type 2 diabetes) (adjusted OR 1.93, 95% CI 1.73–2.14; *P =* 1.06 × 10^−33^).

### Validation cohort

**Fig 2:**
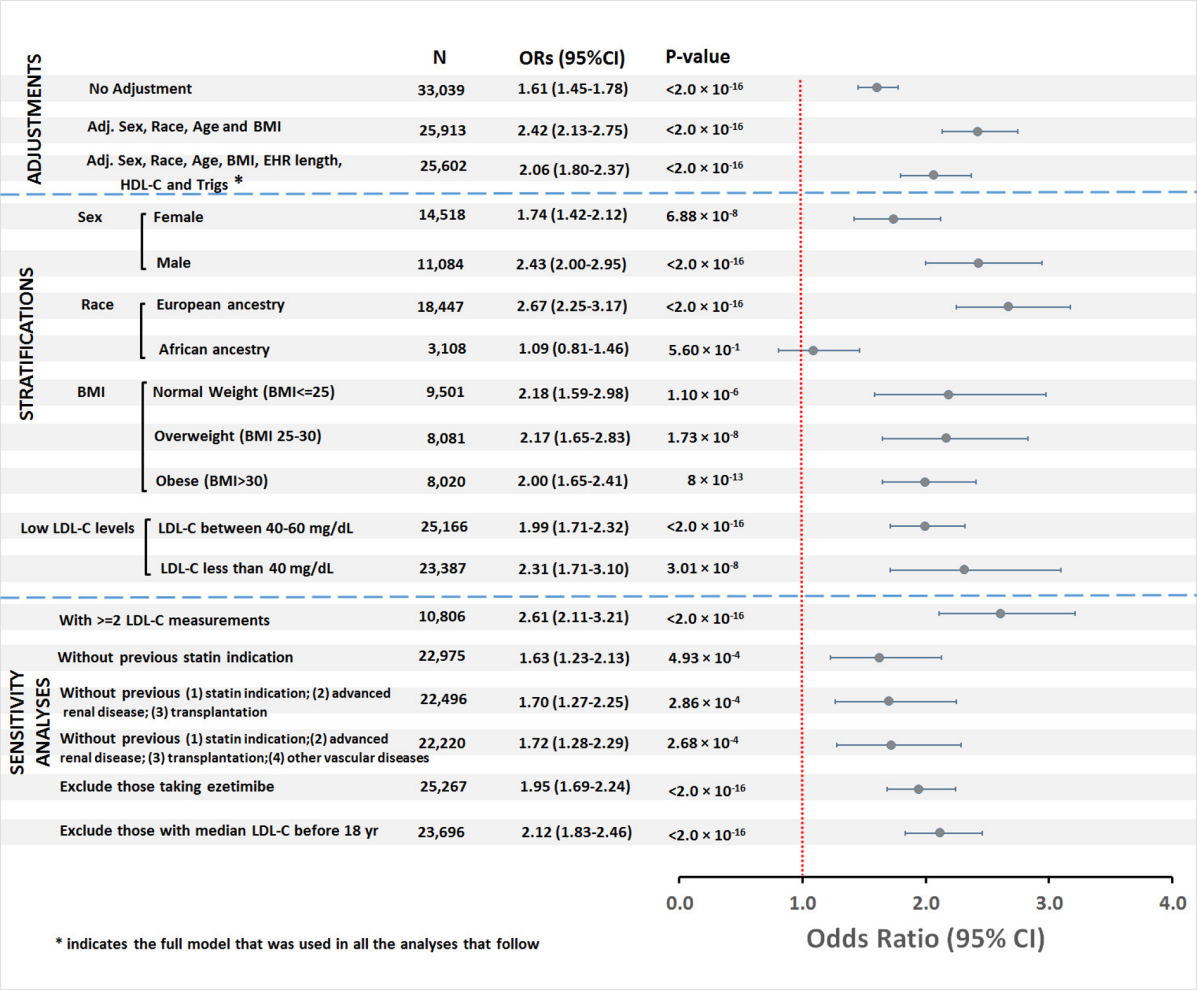
Association between low LDL-C and type 2 diabetes in the validation cohort. BMI, body mass index; EHR, electronic health record; HDL-C, high-density lipoprotein cholesterol; LDL-C, low-density lipoprotein cholesterol; OR, odds ratio; Trigs, triglycerides.

There were 518 cases of T2DM (8.7%) and 2,896 controls in the low LDL-C group, and 2,968 cases of T2DM (6.2%) and 26,657 controls in the normal LDL-C group (S2 Table). The risk of developing T2DM was higher in the low than the normal LDL-C group (OR 1.61, 95% CI 1.45–1.78, *P <* 2.0 × 10^−16^ [unadjusted]; OR 2.42, 95% CI 2.13–2.75, *P <* 2.0 × 10^−16^ [adjusted for age, race, sex, and BMI,]; and OR 2.06, 95% CI 1.80–2.37, *P <* 2.0 × 10^−16^ [adjusted for age, race, sex, BMI, length of EHR, HDL-C, and triglycerides]) (Fig 2).

To further explore the role of clinical and demographic variables, we stratified the groups by sex, BMI, race, and LDL-C level. Low LDL-C increased risk of T2DM in men and women and in all BMI strata (normal weight, overweight, and obese) (Fig 2). No significant interaction was observed between low LDL-C and sex (*P =* 0.056), nor between low LDL-C and BMI (*P =* 0.60). However, the interaction effect between low LDL-C and race was significant (*P =* 1.51 × 10^−6^). The odds of developing T2DM in individuals with low compared to normal LDL-C was increased in those of European ancestry (OR 2.67, 95% CI 2.25–3.17; *P <* 2.0 × 10^−16^) but not African ancestry (OR 1.09, 95% CI 0.81–1.46; *P =* 0.56) (Fig 2). In analyses in which the low LDL-C group was stratified into 2 groups, LDL-C < 40 mg/dl and between 40 and 60 mg/dl, both groups had increased risk of T2DM compared to the normal LDL-C group (OR 2.31, 95% CI 1.71–3.10, *P =* 3.01 × 10^−8^, and OR 1.99, 95% CI 1.71–2.32, *P <* 2.0 × 10^−16^, respectively; Fig 2).

Sensitivity analyses restricted to (1) individuals with 2 or more LDL-C measurements; (2a) those without a previous potential indication for a statin, and (2b) without advanced renal disease or organ transplantation, and (2c) without previous stroke, transient cerebral ischemia, or peripheral vascular disease; (3) those not ever exposed to ezetimibe; and (4) excluding those who were <18 years old at the time their median LDL-C measurement was obtained were consistent with the main analysis (Fig 2).

## Discussion

The major new finding of the study is that low LDL-C concentrations occurring independently of statin treatment were associated with a 2-fold increased risk of T2DM.

Elevated circulating LDL-C concentrations are a major risk factor for coronary heart disease and every 1.0-mmol/l (approximately 39 mg/dl) reduction of LDL-C could reduce the incidence by 10%–20% [20,21]. The cardiovascular benefit of statins is generally thought to be proportional to the amount LDL-C is lowered [20]; thus, very low levels of LDL-C may be desirable. However, there have been concerns about the safety of very low levels of LDL-C because cholesterol is critical for maintaining normal cellular functions. With the advent of powerful LDL-C-lowering drugs, such as statins and PCSK9 inhibitors, very low levels of LDL-C are increasingly common in patients. Indeed, in recent trials, PCSK9 inhibitors in combination with statins resulted in 37% of patients reaching LDL-C concentrations of <25 mg/dl [3,22,23]. Therefore, the relationship between risk of T2DM and low LDL-C concentrations is an important concern.

Increasing epidemiologic and clinical trial evidence indicates that statin therapy increases the risk of T2DM. For example, in the Justification for the Use of Statins in Primary Prevention (JUPITER) trial, physician-reported T2DM was more frequent in patients treated with rosuvastatin than placebo [24]. Also, in Mendelian randomization studies, alleles in *HMGCR* and *PCSK9* associated with lower LDL-C concentrations were associated with increased risk of T2DM—a finding directionally consistent with our findings.

We observed a higher risk of T2DM with low LDL-C concentrations than that reported in statin trials. Meta-analyses of large-scale trials found an approximately 9%–12% increased risk of new-onset T2DM in patients treated with statins [4,5]. The higher risk of T2DM in the current study may be due to the large difference in LDL-C concentrations between the low and normal LDL-C groups. The median LDL-C decrement in statin trials was approximately 36 mg/dl, whereas the difference in LDL-C concentrations between the low and normal LDL-C groups in the current study was 57 mg/dl.

In addition to very low LDL-C concentrations, another factor that might contribute to the risk of T2DM being greater in the current study than that observed in statin trials is the long duration of exposure to low LDL-C concentrations. While statin trials thus far have had a relatively short period of follow-up of a few years, the individuals we identified are likely to have had low LDL-C as a lifelong exposure. In keeping with that idea, genetic studies also found a higher risk of T2DM than would have been predicted from the statin trials. A genetic study reported that each 10-mg/dl predicted decrease in LDL-C was associated with an approximately 11% increased risk of T2DM [7]; thus the 57-mg/dl LDL-C difference in the current study would have been predicted to result in an approximately 63% increased risk of T2DM.

In keeping with the idea that markedly lower LDL-C concentrations are associated with greater risk of T2DM is the observation that intensive statin treatment was associated with a higher risk of T2DM compared to moderate-dose treatment [5]. In the JUPITER study, the risk of T2DM was greater in patients who reached an LDL-C < 30 mg/dl than in those with higher levels [25]. Similarly, we observed a higher OR for T2DM in individuals with LDL-C < 40 mg/dl than in those with LDL-C between 40 and 60 mg/dl (2.30 versus 1.99).

The relationship between weight and risk of T2DM has complicated the interpretation of findings in statin trials and Mendelian randomization studies. There is controversy whether the association between low LDL-C and increased risk of T2DM in patients receiving statins is secondary to the small but significant weight gain associated with statin therapy [5]. Additionally, LDL-C-lowering alleles in both *HMGCR* and *PCSK9* that were associated with increased risk of T2DM were also associated with higher weight and greater waist circumference in Mendelian randomization studies [5,8]. Two of our findings suggest that the association between low LDL-C and T2DM is not strongly related higher weight. First, the association between low LDL-C and T2DM was significant with and without statistical adjustment for BMI. Second, the increased risk of T2DM was present in each BMI category (normal, overweight, and obese).

The relationship between sex and risk of T2DM associated with statin use is also of interest; some studies suggest that women are at higher risk [24,26]. For example, Mora et al. reported that 1 year of treatment with rosuvastatin resulted in a higher risk of T2DM in women (hazard ratio 1.49, 95% CI 1.11–2.01; *P =* 0.008) than men (hazard ratio 1.14, 95% CI 0.91–1.43; *P =* 0.24) [24]. In contrast, in the current study, the risk of T2DM associated with low LDL-C was higher in men than women.

An interesting observation was that the risk of T2DM was not increased significantly in individuals of African ancestry with low LDL-C levels. This may be due partly to the higher risk of T2DM observed in individuals of African ancestry with normal LDL-C levels. While individuals of European and African ancestry in the low LDL-C cohort had a similar proportion of T2DM cases (8.6% and 9.2%, respectively), there was a higher proportion of T2DM cases in those of African than European ancestry in the normal LDL-C cohort (10.0% and 5.7%, respectively). Both genetic and environmental factors could contribute to this observation. Although the genetic architecture for circulating lipid levels and changes in lipid levels with statin therapy are well described in populations of European ancestry [27,28], relatively little is known in populations of African ancestry [29]. The underlying genetic mechanisms for low LDL-C and its relationships to diabetes may differ between those of European and African ancestries. Future validation across different races is desirable.

It has been difficult to identify a relationship between low LDL-C concentrations and risk of T2DM because most of the information about this relationship has come from statin studies. In the setting of statin therapy, the increased risk of T2DM could be mediated by an effect unrelated to the LDL-C lowering by statins resulting in increased insulin resistance and impaired β-cell function [30]. However, genetic studies showing that variants in genes other than *HMGCR* that mediate lower LDL-C concentrations through different mechanisms are also associated with T2DM suggest that low LDL-C concentrations per se are important. Support for the hypothesis that LDL-C concentrations affect risk of T2DM comes from reports that patients with familial hypercholesterolemia, a group with very high LDL-C levels, are less likely to develop T2DM than their relatives with normal LDL-C concentrations [31]. Additional support comes from a report of approximately 6,000 individuals in the Framingham Heart Study in whom a higher LDL-C was associated with a lower risk of T2DM [32]. Such observations argue for mechanisms unrelated to isolated genetic or environmental factors that affect LDL-C.

The potential mechanisms whereby long-term low LDL-C concentrations could lead to the development of T2DM are not known. However, levels of cholesterol in pancreatic β-cells play an important role in regulating insulin secretion. Experiments that inhibited cholesterol biosynthesis in pancreatic islet cells showed that the resulting low levels of cellular cholesterol were associated with impaired insulin secretion, and these effects were reversed by cholesterol repletion [33]. Additional experiments will be required to further elucidate the underlying mechanisms.

There are several advantages to performing studies in large EHRs including the ability to study large numbers of patients and obtain information about drugs, diagnoses, and laboratory values longitudinally. However, there are also several limitations. Misclassification can occur due to EHR fragmentation. Thus, a patient could receive care from multiple healthcare providers, and consequently a complete statin exposure history, for example, might not be captured. We conducted 2 sensitivity analyses to address this possibility. First, we excluded individuals with a previous illness representing a possible indication for a statin (diabetes or myocardial infarction), and, second, we restricted the analysis to individuals with 2 or more LDL-C measurements. Because medications are recorded at each visit, requiring measurement of LDL-C on at least 2 occasions would make it less likely that statin use had not been captured. Additionally, our eligibility criteria for the low LDL-C group excluded individuals with any LDL-C measurement ever ≥80 mg/dl. Thus, individuals in whom a statin was started and this was not captured were likely to have been excluded based on a previous high LDL-C measurement.

Since the study cohort was not an inception cohort, another limitation is that we cannot exclude the possibility of reverse causation (i.e., T2DM, or factors that cause T2DM, result in a low LDL-C). This seems unlikely since T2DM is not a recognized cause of low LDL-C, and Mendelian randomization predictors of low LDL-C are associated with increased risk of T2DM. Also, to address this question, we performed sensitivity analyses restricted to individuals in whom T2DM was diagnosed after the initial low LDL-C, and that excluded individuals with chronic illness associated with diabetes such as renal failure and organ transplantation. Low LDL-C remained a significant predictor for T2DM in these analyses. A third limitation is that we did not perform genetic analyses to identify the relationship between specific variants, LDL-C, and risk of T2DM. Genome-wide genotype information is not currently available for the majority of the study participants. We do not know the exact causes of low LDL-C in this population, and some of the variation is likely due to genetic factors. However, meta-analyses of genetic risk variants in large global consortia have found that only approximately 15% of variability in LDL-C levels is explained by known genetic risk variants [27,28]. It is likely that the genetic component of low LDL-C concentrations is due to many variants, some as yet unidentified. Another limitation of the study is that the majority of LDL-C measurements used were calculated using the Friedewald equation because direct LDL-C measurements are seldom performed in routine clinical practice. A disadvantage of the Friedewald equation is that it can underestimate LDL-C levels in the presence of elevated triglyceride levels, as is more likely to occur in patients with diabetes. However, analyses that adjusted for triglyceride levels did not alter our findings materially. Prospective studies with direct measurement of LDL-C concentration or large Mendelian randomization studies would address the potential for reverse causality between T2DM and low LDL-C levels.

A final limitation is that we only compared groups with low and normal LDL-C levels. This design was chosen intentionally to be relevant to the clinical situation in which patients might receive therapy of varying intensity to lower LDL-C. A limitation of the design is that we do not have information about the risk of T2DM in individuals with higher LDL-C concentrations.

### Conclusions

Low circulating LDL-C levels unrelated to statin use were associated with increased risk of T2DM in individuals of European ancestry in this medical-center-based observational study. Longitudinal cohort studies to assess the relationship between low LDL-C levels not associated with lipid-lowering therapy and risk of developing T2DM will be important.

## Supporting information

S1 RECORD Checklist RECORD checklist. (DOCX)

S1 Fig Flowchart for patient identification in the sensitivity analyses. (TIF)

S1 Table Mapping between phecode and ICD-9 code. (DOCX)

S2 Table Demographic characteristics for validation cohort. (DOCX)

S3 Table Association between low LDL-C and type 2 diabetes phecodes (discovery phase). (DOCX)

## Abbreviations

BMI: body mass index
EHR: electronic health record
HDL-C: high-density lipoprotein cholesterol
LDL-C: low-density lipoprotein cholesterol
OR: odds ratio
T2DM: type 2 diabetes mellitus
