# Supplementary figures and images for: Relationship between very low low-density lipoprotein cholesterol concentrations not due to statin therapy and risk of type 2 diabetes: A US-based cross-sectional observational study using electronic health records
Source: PLoS Med. 2018 Aug 28;15(8):e1002642. doi: 10.1371/journal.pmed.1002642 (PMC6112635; doi:10.1371/journal.pmed.1002642)

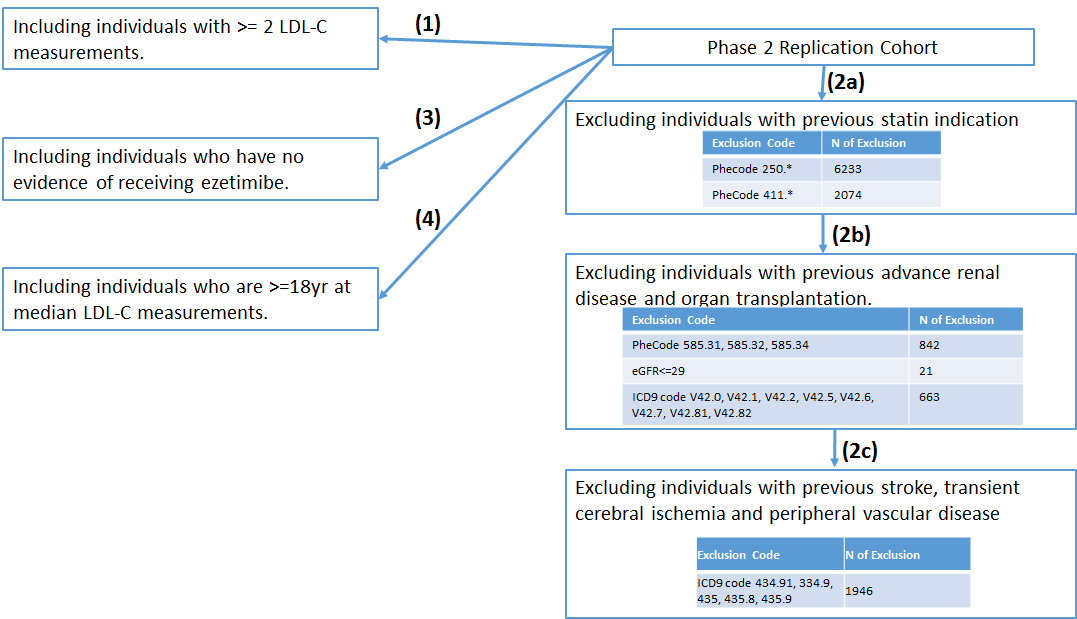

Supplement: S1 Fig — (TIF) [file pmed.1002642.s002.tif]
